# *Azohydromonas caseinilytica* sp. nov., a Nitrogen-Fixing Bacterium Isolated From Forest Soil by Using Optimized Culture Method

**DOI:** 10.3389/fmicb.2021.647132

**Published:** 2021-05-21

**Authors:** Ram Hari Dahal, Dhiraj Kumar Chaudhary, Dong-Uk Kim, Jaisoo Kim

**Affiliations:** ^1^Department of Life Sciences, College of Natural Sciences, Kyonggi University, Suwon-si, South Korea; ^2^Department of Microbiology, School of Medicine, Kyungpook National University, Daegu, South Korea; ^3^Department of Environmental Engineering, Korea University Sejong Campus, Sejong City, South Korea; ^4^Department of Biological Science, College of Science and Engineering, Sangji University, Wonju-si, South Korea

**Keywords:** *Azohydromonas caseinilytica* sp. nov., forest soil, N_2_-fixation, CO_2_ assimilation, next generation sequencing, uncultured bacterium

## Abstract

A bacterial strain, designated strain G-1-1-14^T^, was isolated from Kyonggi University forest soil during a study of previously uncultured bacterium. The cells of strain G-1-1-14^T^ were motile by means of peritrichous flagella, Gram-stain-negative, rod-shaped, and able to grow autotrophically with hydrogen and fix nitrogen. Phylogenetic analysis based on 16S rRNA gene sequence indicated that strain G-1-1-14^T^ belonged to the genus *Azohydromonas*. The closest species of strain G-1-1-14^T^ were *Azohydromonas ureilytica* UCM-80^*T*^ (98.4% sequence similarity), *Azohydromonas lata* IAM 12599^*T*^ (97.5%), *Azohydromonas riparia* UCM-11^*T*^ (97.1%), and *Azohydromonas australica* IAM 12664^*T*^ (97.0%). The genome of strain G-1-1-14^T^ was 6,654,139 bp long with 5,865 protein-coding genes. The genome consisted of N_2_-fixing genes (*nifH*) and various regulatory genes for CO_2_ fixation and H_2_ utilization. The principal respiratory quinone was ubiquinone-8, and the major polar lipids were phosphatidylethanolamine, diphosphatidylglycerol, and phosphatidylglycerol. The major fatty acids were summed feature 3 (iso-C_15__:__0_ 2-OH and/or C_16__:__1_*ω*7*c*), C_16__:__0_, summed feature 8 (C_18__:__1_*ω*7*c* and/or C_18__:__1_*ω*6*c*), and cyclo-C_17__:__0_. The DNA G + C content was 69.9%. The average nucleotide identity (OrthoANI), *in silico* DNA–DNA hybridization (dDDH), and conventional DDH relatedness values were below the species demarcation values for novel species. Based on genomic, genetic, phylogenetic, phenotypic, and chemotaxonomic characterizations, strain G-1-1-14^T^ represents a novel species within the genus *Azohydromonas*, for which the name *Azohydromonas caseinilytica* sp. nov. is proposed. The type strain is G-1-1-14^T^ (= KACC 21615^*T*^ = NBRC 114390^*T*^).

## Introduction

Metagenomic, next-generation sequencing, and whole-genome sequencing technology help to reclassify and correct the taxonomic position of bacterial species in systematic studies. In recent years, various taxa have been reclassified based on 16S ribosomal RNA (rRNA) gene and whole-genome analysis, and genera are more complete than they previously were. Similarly, the genus *Azohydromonas* was first proposed by reclassifying the genus [*Alcaligenes*] (Xie and Yokota, 2005). On the other hand, these technologies revealed that most environmental microorganisms are still “not-yet cultured” on synthetic media and comprise around 99% of microorganisms previously uncultured (Vartoukian et al., 2010; Chaudhary et al., 2019). The simple to complex reasons for failing to cultivate these uncultivated microorganisms include the lack of correct nutrients, use of nutrient-rich media, unsuitable pH, insufficient incubation time, inappropriate temperature, need of specific growth signal, dependence on other microorganisms, or failure to maintain a natural growth environment (Vartoukian et al., 2010; Pulschen et al., 2017; Chaudhary et al., 2019). We have isolated a bacterium, designated strain G-1-1-14^T^, from Kyonggi University forest soil using low nutrient and long enrichment time. In addition, the isolated strain showed best match with a previously uncultured bacterium (non-type material sequences) within Megablast (GenBank) and belonged to the genus *Azohydromonas*.

Currently, the genus *Azohydromonas* comprise only five species – *Azohydromonas lata*, *Azohydromonas australica*, *Azohydromonas riparia*, and *Azohydromonas ureilytica* (Xie and Yokota, 2005; Nguyen and Kim, 2017) – with validly published names in addition to “*Azohydromonas aeria*” (Xue et al., 2020), which has not been validated yet. Members of the genus *Azohydromonas* are characterized as motile by means of flagella, non-spore-forming, Gram-stain-negative, rod- or coccid-shaped, DNA G + C content between 69 and 72 mol%, ubiquinone-8 (Q-8) as the major respiratory quinone, summed feature 3 (iso-C_15__:__0_ 2-OH and/or C_16__:__1_*ω*7*c*), C_16__:__0_, and summed feature 8 (C_18__:__1_*ω*7*c* and/or C_18__:__1_*ω*6*c*) as the principal fatty acids in addition to cells accumulating PHB (poly-β-hydroxybutyrate) granules as storage material (Xie and Yokota, 2005; Nguyen and Kim, 2017). The members are able to fix nitrogen and grow autotrophically with hydrogen (Xie and Yokota, 2005). This study describes the polyphasic study and taxonomic position of strain G-1-1-14^T^ isolated from forest soil.

## Materials and Methods

### Isolation and Ecology

Strain G-1-1-14^T^ was isolated from Kyonggi University forest soil, geographically located at Suwon-si, Gyeonggi-do, South Korea (37°18′5″ N and 127°1′56″ E). The pH of the soil was 6.8. A modified culture method using a six-well Transwell plate (Corning Inc., NY, United States) was used for isolation. Debris-free sieved soil (∼3 g) was kept on the bottom of the Transwell plate and 3 ml of diluted (1/10) Reasoner’s 2A (R2A) broth (MBcell; KisanBio, Seoul, South Korea) was added to the insert. After that, 100 μl of soil suspension (1 g of soil in 9 ml of distilled water) was added to the insert. Then, the Transwell plate was incubated in a shaker at 130 rpm at 28°C for 6 weeks. After 6 weeks, enriched culture was serially diluted and then 100 μl of each dilution was spread on 1/10 R2A agar plates (the agar used in this research was Agar A; Bio Basic, Toronto, ON, Canada) and incubated at 28°C for 4 weeks (Dahal and Kim, 2018c). The short-term maintenance and the long-term preservation of the isolate were done as described previously (Dahal et al., 2018).

### 16S rRNA Phylogeny

The genomic DNA of strain G-1-1-14^T^ was extracted using InstaGene Matrix kit (Bio-Rad, Hercules, CA, United States) according to the manufacturer’s instruction. Amplification of 16S rRNA gene was done by PCR using primers 27F and 1492R (Frank et al., 2008). Sequencing was carried out using a 3770XL DNA analyzer with a BigDye Terminator cycle sequencing kit v.3.1 (Applied Biosystems, CA, United States). Near-complete sequences of the 16S rRNA genes were assembled with SeqMan software (DNASTAR, Inc., Madison, WI, United States). For type material sequences, the closest phylogenetic neighbors were identified by using the EzBioCloud server (Yoon et al., 2017b). In addition, non-type material sequences were compared with the top hits of Megablast (GenBank). All the 16S rRNA gene sequences of the closest phylogenetic members were retrieved from the whole-genome sequences (WGS) and/or GenBank database and aligned using SINA^1^ (Pruesse et al., 2012). Phylogenetic trees were reconstructed using MEGA7 (Kumar et al., 2016). Neighbor-joining and maximum-likelihood trees were reconstructed using the Kimura two-parameter model based on 1,000 bootstrap replications and partial deletion with 95% site coverage cutoff (Felsenstein, 1981; Saitou and Nei, 1987). In addition, maximum parsimony tree was inferred with the “Mini-Mini Heuristic” search method.

### Genome Features

Whole-genome-based approaches were used for further analysis of the taxonomic status of the novel strain. For whole-genome sequencing, the genomic DNA was extracted by using DNeasy Blood and Tissue kits (Qiagen). Whole-genome shotgun sequencing of strain G-1-1-14^T^ was performed by Macrogen (Seoul, South Korea) using the Illumina HiSeq platform and assembled by SPAdes (Bankevich et al., 2012). The authenticity of the genome assembly was checked by comparing the 16S rRNA gene sequences using the NCBI Align Sequences Nucleotide BLAST tool (Zhang et al., 2000) and the potential contamination was checked by ContEst16S algorithm (Lee et al., 2017). After analysis, the whole-genome sequence was annotated using NCBI Prokaryotic Genome Annotation Pipeline (PAPG) (Tatusova et al., 2016) and Rapid Annotations using the Subsystems Technology (RAST) server (Aziz et al., 2008). The 92 core genes were extracted from genomes using Prodigal v2.6.3 (Hyatt et al., 2010) and hmmsearch v3.1b2 (Eddy, 2011). The amino acid sequences of 92 core genes were aligned by using MAFFT 7.310 (Katoh and Standley, 2013) and concatenated into a single alignment. The alignment position that had a gap >50% were excluded. Then, the phylogenomic tree was inferred by using FastTree (Price et al., 2010) and RAxML (Stamatakis, 2014) and viewed using MEGA X v10.1 (Kumar et al., 2018). The branch support inference was based on 100 non-parametric bootstrap replicates, and the branch supports of the phylogenomic tree were evaluated using gene support index (GSI). The genome-based relatedness between strain G-1-1-14^T^ and the phylogenetically related type strains was determined based on average nucleotide identity (ANI) using the OrthoANI algorithm (Yoon et al., 2017a). The digital DNA–DNA hybridization (dDDH) was calculated *in silico* by the Genome-to-Genome Distance Calculator using the blast method (Meier-Kolthoff et al., 2013). In addition, conventional DDH was measured fluorometrically using photobiotin-labeled DNA probes and microdilution plates as recommended by Ezaki et al. (1989). Moreover, annotation and analysis of the secondary metabolite biosynthesis genes were carried out by using the antiSMASH server (Blin et al., 2019). The COG (Clusters of Orthologous Group) functional categories were assigned by searching against the KEGG (Kyoto Encyclopedia of Genes and Genomes) database (Kanehisa and Goto, 2000). Furthermore, the CRISPR gene and Cas cluster were analyzed using the CRISPRCasFinder online server^2^.

#### Physiology and Chemotaxonomy

The cell morphology of strain G-1-1-14^T^, grown on R2A agar for 5 days at 28°C, was examined by transmission electron microscopy (Talos L120C, FEI). Colony morphology was observed by a Zoom Stereo Microscope (SZ61; Olympus, Tokyo, Japan). Gram staining was performed as described previously (Doetsch, 1981). Motility was tested in the R2A medium containing 0.4% (*w*/*v*) agar. Oxidase activity was determined using 1% (*w*/*v*) *tetra*-methyl-*p*-phenylenediamine dihydrochloride. Catalase activity was assessed using 3% (*v*/*v*) hydrogen peroxide (H_2_O_2_). Growth at various temperatures (4–50°C) on R2A agar plates was observed for 10 days. Growth was determined on various media including tryptone soya agar (TSA; Oxoid), R2A agar, nutrient agar (NA; Oxoid), sorbitol MacConkey agar (MA; Oxoid), potato dextrose agar (PDA; Becton), marine agar 2216 (Becton), brain heart infusion (BHI) agar (Oxoid), veal infusion agar (Becton), and Luria–Bertani agar (LBA; Oxoid). The salt tolerance of strain G-1-1-14^T^ was examined in R2A broth supplemented with NaCl (0–5%, *w*/*v*, at 0.5% intervals). The pH range for growth was determined by cultivation at 28°C in R2A broth adjusted to pH 4–12 (at 0.5 pH unit increment) prior to sterilization using citrate/NaH_2_PO_4_ buffer (for pH 4.0–5.5), phosphate buffer (for pH 6–7.5), Tris buffer (for pH 8–10) and 5 M NaOH (for pH 10.5–12.0) (Dahal and Kim, 2018b). The hydrolysis of Tween 80, Tween 60, and Tween 40 was assessed using the method of Smibert and Krieg (1994). Anaerobic growth was examined on R2A agar at 28°C for 10 days by using the BD GasPak EZ Gas Generating Pouch System. The hydrolysis of starch, chitin, carboxymethyl (CM)-cellulose, tyrosine, and casein was examined as previously described (Dahal and Kim, 2018a). A DNase activity assay was performed with DNase agar (Oxoid). The presence of spores was examined by staining with malachite green. Autotrophic growth on hydrogen and nitrogen fixation was done as recommended by Pedrosa et al. (1980). For the nitrogen fixation test, strain G-1-1-14^T^ was grown on N-free semi-solid (NFb) medium and N-free agar plate with a bromothymol blue indicator (da Silva Lima et al., 2014). In addition, for autotrophic growth in hydrogen, the culture was supplied with 10% O_2_, 10% CO_2_, 20% N_2_, and 60% H_2_ (*v*/*v*) in NFb medium. Other physiological tests were performed using API 20NE and API ID 32GN kits (bioMérieux, Marcy-l’Étoile, France). Enzyme activities were observed using an API ZYM kit (bioMérieux) as per the manufacturer’s instructions.

For fatty acid analysis, the cells of strain G-1-1-14^T^ and the reference strains were harvested from the same culture condition during the late log phase (at 28°C for 4 days on R2A agar plate). The cellular fatty acids were extracted by using the MIDI protocol (Sherlock Microbial Identification System, version 6.0B), analyzed with a gas chromatograph (GC; HP 6890 Series GC System, Hewlett Packard), and identified using the TSBA6 database of the Microbial Identification System (Sasser, 1990). Polar lipids and isoprenoid quinones were extracted from freeze-dried cells according to the procedures described by Minnikin et al. (1984). Appropriate detection reagents were used to identify the spots (Komagata and Suzuki, 1988).

## Results and Discussion

### Phylogenetic Analysis

**FIGURE 1:**
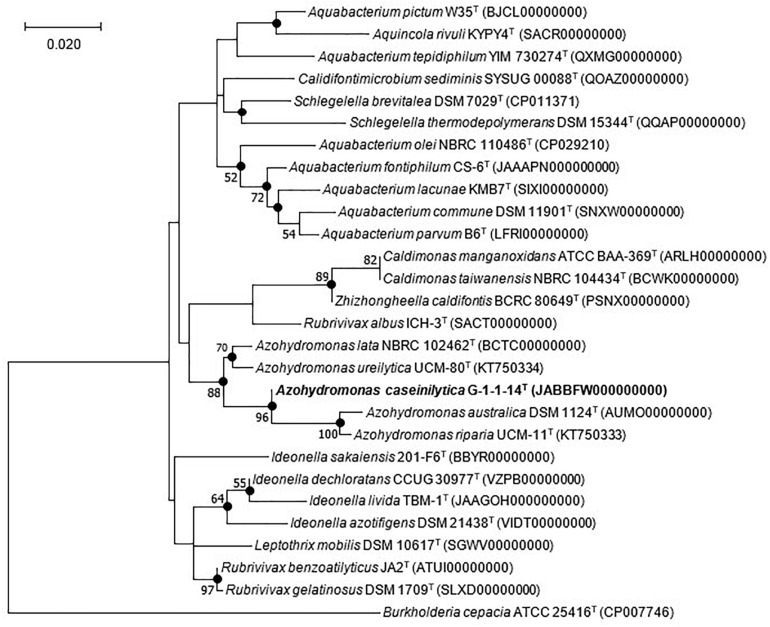
Maximum likelihood tree based on 16S rRNA gene sequences showing the phylogenetic position of strain G-1-1-14^T^ among the closely related members of the order Burkholderiales. *Filled circles* indicate nodes recovered by all three treeing methods (neighbor-joining, maximum likelihood, and maximum parsimony). The *numbers at the nodes* indicate the percentage of 1,000 bootstrap replicates yielding this topology; only values >50% are shown. *Burkholderia cepacia* ATCC 25416^*T*^ was used as an outgroup. The GenBank accession numbers are given in *parentheses*. *Bar*, 0.020 substitution per nucleotide position. Bold values indicates proposed species.

The nucleotide sequence of the 16S rRNA gene of strain G-1-1-14^T^ has been deposited in the GenBank/EMBL/DDBJ database under the accession MN685324. Preliminary comparisons with the 16S rRNA gene sequences in GenBank showed top hits with previously uncultured bacterial clones (Supplementary Figure 1). Among the five closely related uncultured bacterial clones, TSNR003_118, TSSUR003_P21, and TS8 have been isolated from rice paddy soil, whereas bacterial clones BJ201307-105 and SH201208-30 are from rainwater. These results showed that the closest members of strain G-1-1-14^T^ could be isolated not only from the soil but also from rainwater. In addition, the 16S rRNA gene sequence of strain G-1-1-14^T^ was analyzed with the EzBioCloud server against the type strain sequences. Strain G-1-1-14^T^ belonged to the family *Alcaligenaceae* of the order *Burkholderiales* and was most closely related to *A. ureilytica* UCM-80^*T*^ (98.4% sequence similarity), *A. lata* IAM 12599^*T*^ (97.5%), *A. riparia* UCM-11^*T*^ (97.1%), and *A. australica* IAM 12664T (97.0%). Strain G-1-1-14^T^ was well clustered with the other members of the genus *Azohydromonas* in the neighbor-joining (NJ), maximum likelihood (ML), and maximum parsimony (MP) trees (Figure 1 and Supplementary Figures 2, 3). In addition, a monophyletic clade formed within the genus *Azohydromonas* with a strong bootstrap value validated for strain G-1-1-14^T^ as a novel member of genus *Azohydromonas* (Figure 1).

For physiology, biochemical, quinone, and fatty acid analyses, the reference strains *A. ureilytica* UCM-80^*T*^, *A. lata* KACC 15149^*T*^, *A. riparia* UCM-11^*T*^, and *A. australica* KACC 15148^*T*^ were selected and analyzed under identical conditions.

### Genome Analysis

**FIGURE 2:**
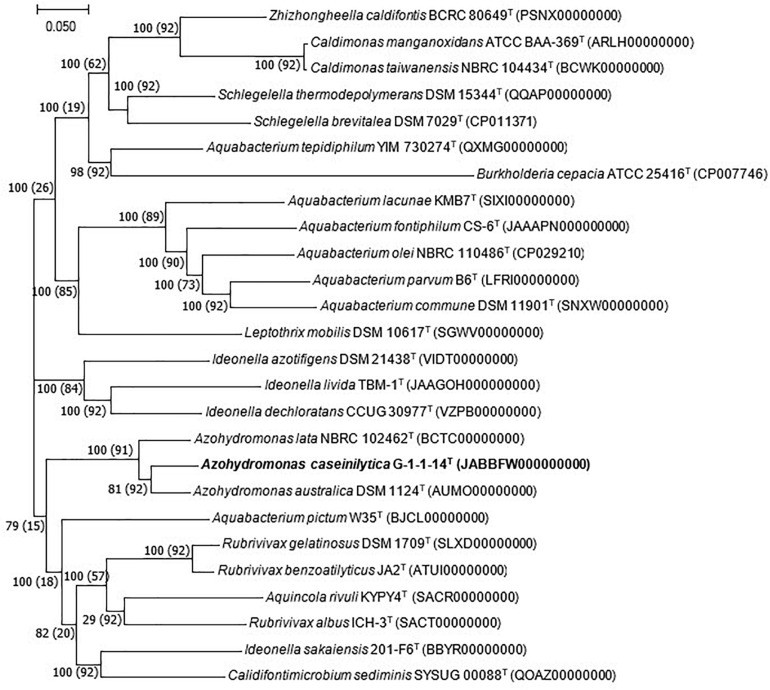
Phylogenomic tree based on the concatenated alignment of 92 core genes. *Bar*, 0.050 substitution per nucleotide position. Bootstrap and gene support index (GSI) values are shown in the *branch nodes*. Values in *parentheses* are GSI. Bold values indicates proposed species.

The whole-genome shotgun sequence of strain G-1-1-14^T^ has been deposited at DDBJ/ENA/GenBank under the accession JABBFW000000000. The whole-genome sequence of strain G-1-1-14^T^ was 6,654,139 bp long with DNA G + C of 69.9%. The genome was assembled in 98 scaffolds with an N50 value of 195,393 bp, 351 subsystems, and genome coverage of 162.0× (Table 1). The genome features of the type species of *A. lata* and *A. australica* are also presented in Table 1. The DNA G + C content was calculated based on whole-genome sequences. The OrthoANI values of strain G-1-1-14^T^ for *A. lata* NBRC 102462^*T*^, *A. australica* DSM 1124^*T*^, and “*A. aeria*” t3-1-3 were 84.6, 86.5, and 85.3%, respectively (Supplementary Table 1). Similarly, the *in silico* DDH values for *A. lata* NBRC 102462^*T*^, *A. australica* DSM 1124^*T*^, and “*A. aeria*” t3-1-3 were 28.6, 31.5, and 29.7, respectively (Supplementary Table 1). These obtained values were below the threshold ANI value of 95.0–96.0% and dDDH value of <70% used for species delineation (Richter and Rosselló-Móra, 2009; Meier-Kolthoff et al., 2013). The DNA–DNA hybridization of strain G-1-1-14^T^ with reference strains *A. riparia* UCM-11^*T*^ and *A. ureilytica* UCM-80^*T*^ showed DDH relatedness of 45.8 ± 2.7% and 31.4 ± 2.6%, respectively. The DNA–DNA relatedness between these species clearly showed that strain G-1-1-14^T^ differs genetically from the *Azohydromonas* type strains at the species level (Wayne et al., 1987). In addition, the phylogenomic tree constructed using concatenated 92 core genes also proved that strain G-1-1-14^T^ is a novel member of the genus *Azohydromonas* (Figure 2). Moreover, due to the unavailability of the genome data of *A. ureilytica* UCM-80^*T*^ and *A. riparia* UCM-11^*T*^, these strains were not used in the phylogenomic tree reconstruction. However, strain G-1-1-14^T^ well clustered with the other members of the genus *Azohydromonas* and distinguished with other clusters (Figure 2). The phylogenomic inference is supported by high bootstrap and GSI values. Furthermore, multilocus sequence analysis (MLSA) trees based on the *rpoB* and *recA* genes showed high robustness of strain G-1-1-14^T^ with the *Azohydromonas* members (Supplementary Figures 4, 5).

**TABLE 1:** Genome features of strain G-1-1-14^T^, *Azohydromonas australica* DSM 1124^*T*^, and *Azohydromonas lata* NBRC 102462^*T*^.

Genome features	Value		
	G-1-1-14^T^	*A. australica* DSM 1124^*T*^	*A. lata* NBRC 102462^*T*^
Genome size (bp)	6,654,139	8,749,129	7,183,251
G + C content (%)	69.9	68.7	69.0
No. of contigs	98	142	342
N50	195,393	115,405	53,184
No. of subsystem	351	364	347
No. of proteins	5,830	7,662	6,292
Total genes	6,029	8,070	6,528
CDSs (total)	5,955	7,662	6,292
Genes (RNA)	73	71	54
rRNAs (5S, 16S, 23S)	8 (4, 3, 1)	15	4
tRNAs	60	52	46
ncRNAs	5	4	4
Pseudo genes (total)	90	337	182
CRISPR repeats	10	16	7
Cas cluster	3	4	9
Genome coverage	162.0×	Unknown	114.0×
N_2_ fixation genes	*ptsN*, *frdN, nifA, nifB, frdN, nifX, nifX2, nifE, nifN, nifQ, nifV, nifW, nifH, nifD, nifK, nifZ, nifT, nifO*	*ntrC, fixC, nifA, iscA-like, nifB, frdN, nifX, nifX2, nifE, nifN, nifQ, nifV, nifW, nifH, nifD, nifK, nifZ, nifT, nifO*	*ptsN*, *nifT, nifA, iscA-like, nifB, frdN, nifX, nifX2, nifE, nifN, nifQ, nifV, nifW, nifH, nifD, nifK, nifZ, nifT, nifO*
Metal-resistant genes	*zraR, cusB, czsB, hmrR*	*czcD, czcA, cusB, czsB, cusA, cusR, hmrR*	*czcC, cusR, hmrR*
CO_2_ fixation genes	*rbcS*, *rbcL, hatR*	*rbcS*, *rbcL*	*rbcS, rbcL, hatR*

The genome of strain G-1-1-14^T^ contains nitrogen fixation regulatory genes (*nif* genes) such as *nifA*, *nifB*, *nifD*, *nifE*, *nifH*, *nifK*, *nifN*, *nifO*, *nifQ*, *nifT*, *nifV*, *nifW*, *nifX*, *nifX2*, and *nifZ* (Supplementary Tables 2, 3). Similar nitrogen fixation regulatory genes were also present in the genomes of *A. australica* DSM 1124^*T*^ and *A. lata* NBRC 102462^*T*^. The presence of these genes showed that strain G-1-1-14^T^ fixes atmospheric nitrogen. In addition, the genome showed each chain (small and large) of ribulose 1,5-bisphosphate (RuBP) carboxylase, which is an enzyme in the first principal step of carbon fixation. Moreover, the genome consists of the high-affinity carbon uptake protein (*hat*/*hatR*), which also regulates CO_2_ fixation. The genome consists of the genes (*hypF*, *hypA*, *hypC*, *hypB*, *hypD*, and *hypE*) associated with the [NiFe]-hydrogenase regulatory proteins that are responsible for the uptake of H_2_ gas as a source of energy. Furthermore, the genome contains the genes (*hoxA*, *hoxC*, *hoxO*/*hyaE*, *hoxB*, *hoxT*/*hybE*, and *hypC*/*hoxL*) responsible for hydrogen-sensing regulatory systems in addition to the hydrogenase structural genes (*hupV*, *hupU*, and *hupF*) responsible for hydrogenase activity. These results, along with the data of nitrogen fixation and autotrophic growth assessment, proved that strain G-1-1-14^T^ fixes nitrogen and grows autotrophically in the presence of H_2_ gas. Moreover, other members of the genus *Azohydromonas* have also been reported for autotrophic growth (Xie and Yokota, 2005; Nguyen and Kim, 2017).

Several members of *Azohydromonas* are well-known nitrogen fixers (Xie and Yokota, 2005). Biological nitrogen fixation (BNF) is a key step in the nitrogen cycle as it transforms atmospheric nitrogen into ammonium (Pereira e Silva et al., 2013). The nitrogen cycle and the related microorganisms play crucial roles in the ecosystem, affecting both the agricultural field and climate sectors (Khanal and Lee, 2020). Strain G-1-1-14^T^ showed a direct association with the nitrogen cycle as it reported the ability to fix atmospheric nitrogen. In this regard, this strain can play a valuable ecological role that contributes to maintaining the nitrogen cycle in the environment. Furthermore, strain G-1-1-14^T^ grows autotrophically with the help of H_2_ gas. The genome of this strain is also reported to contain the genes *hat/hatR*, which regulate CO_2_ fixation. CO_2_ fixation is an important phenomenon in the carbon cycle that reduces the CO_2_ emission in the atmosphere. CO_2_ is a primary greenhouse gas that is responsible for global warming (Salehizadeh et al., 2020). Any attempt of reducing CO_2_ emission in the atmosphere can be a milestone to protecting the natural ecosystem. In addition, strain G-1-1-14^T^ produces P3(HB) (and regulatory proteins WP_169159239, WP_169160336, and WP_169163299; Supplementary Figure 6), which is also responsible for reducing the CO_2_ releases in the atmosphere (Zafar et al., 2014). Species of *Azohydromonas* have been revealed to synthesize various poly-β-hydroxyalkanoates (PHAs) (Zafar et al., 2014; Sharma et al., 2017). PHAs have been widely applied for the production of biodegradable and biocompatible plastics (Zafar et al., 2014). From this point of view, strain G-1-1-14^T^ can be considered as a promising bioresource to mitigate plastic-associated problems and environmental issues. Overall, strain G-1-1-14^T^ can play a key role in the both nitrogen and carbon cycles, suggesting that this strain has a significant potential ecological role in the natural habitat.

**FIGURE 3:**
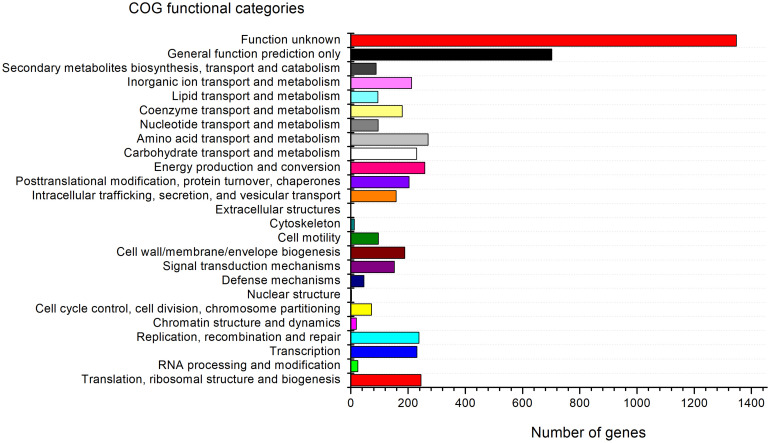
Clusters of Orthologous Group (COG) functional classification of proteins in the genome of strain G-1-1-14^T^.

The genome of strain G-1-1-14^T^ consists of 13 putative biosynthetic gene clusters (BGCs) that are responsible for various secondary metabolites including terpene, burkholderic acid, bacteriocin, hserlactone, aryl polyene, lanthipeptide, non-ribosomal peptide synthetase (NRPS), NRPS-like, and *N*-acetylglutaminylglutamine amide (NAGGN) (Supplementary Table 4). The predicted secondary metabolites may have potential ecological roles, such as hserlactone, which may be related to the communication between fungi and bacteria (Shiner et al., 2005), and aryl polyene, which could protect bacteria from reactive oxidation (Schöner et al., 2016). In addition, RAST analyses revealed the presence of metabolic genes for thiazole/oxazole-modified microcin synthesis, alkaloid biosynthesis, and auxin biosynthesis. Furthermore, DNA metabolism, nitrogen metabolism, carbohydrate metabolism, CO_2_ fixation, phosphorus metabolism, and other metabolic as well as physiologically relevant genes were also present in the genome of strain G-1-1-14^T^ (Supplementary Figure 7). Moreover, 88 genes for secondary metabolite biosynthesis, transport, and catabolism, 1,347 genes with unknown functions, 258 genes for energy production and conversion, and 212 genes for inorganic ion transport and metabolism have been detected in COG functional categories (Figure 3).

### Phenotypic and Chemotaxonomic Characteristics

The cells of strain G-1-1-14^T^ (Supplementary Figure 6) were rod-shaped, Gram-stain-negative, aerobic, and motile by means of peritrichous flagella. The cells were able to grow on N_2_-free semi-solid medium supplemented with 10% O_2_, 10% CO_2_, 20% N_2_, and 60% H_2_ (*v*/*v*) (OD_600_ = 0.3). The cells were 2.6–2.9 μm long and 1.2–1.7 μm wide. Colonies on R2A agar were straw white in color, circular, entire, and convex. The differential phenotypic characteristic features of strain G-1-1-14^T^ are presented in Table 2 with all the type species of the genus *Azohydromonas*. Having the highest salt tolerance (4.0%); casein hydrolysis; assimilation of 3-hydroxybutyric acid, D-ribose, lactic acid, *N*-acetyl-glucosamine, propionic acid, salicin, and valeric acid; and the positive activities of trypsin, valine arylamidase, α-chymotrypsin, α-galactosidase, and β-glucosidase clearly distinguished strain G-1-1-14^T^ from the other type strains (Table 2).

**TABLE 2:** Phenotypic characteristics that differentiate strain G-1-1-14^T^ from the type strains of the genus *Azohydromonas*.

Characteristic	1	2	3	4	5	6^*a*^
Maximum growth temperature (°C)	45	40	40	40	45	42
Highest salt tolerance (%, *w*/*v*)	4.0	3.0	2.0	1.5	2.5	2.0
pH range	5.0–11.0	5.0–9.5	5.0–10.5	5.5–9.5	5.5–9.0	4.0–9.0
Hydrolysis of						
Casein	+	−	−	−	−	ND
Starch	−	−	+	−	−	+
Aesculin	w	−	+	−	+	−
Gelatin	−	−	+	−	−	−
Enzyme activity (API ZYM)						
Cystine arylamidase	+	−	−	−	−	+
Esterase (C4)	w	+	+	+	w	+
Esterase lipase (C8)	+	+	+	+	+	+
Lipase (C14)	−	−	−	−	−	+
Naphthol-AS-BI-phosphohydrolase	w	−	−	−	−	+
Trypsin	+	−	−	−	−	+
Valine arylamidase	+	−	−	−	−	+
α-chymotrypsin	+	−	−	−	−	+
α-galactosidase	+	−	−	−	−	+
α-glucosidase	−	−	+	+	w	+
β-galactosidase	−	−	−	−	−	+
β-glucosidase	+	−	−	−	−	−
β-glucuronidase	−	−	−	−	−	+
Assimilation of						
D-glucose	+	−	+	−	+	w
D-maltose	−	−	+	−	−	−
D-mannitol	−	+	+	−	+	−
D-ribose	+	−	−	−	−	ND
L-alanine	+	−	+	−	−	ND
L-arabinose	−	−	+	−	−	−
L-fucose	−	−	−	−	−	+
L-rhamnose	+	−	−	−	+	+
L-serine	+	−	+	−	−	ND
Malic acid	−	−	−	−	−	+
Potassium gluconate	+	−	+	−	+	w
Salicin	+	−	−	−	−	ND
Trisodium citrate	−	−	−	−	−	+
Valeric acid	+	−	−	−	−	ND
DNA G + C content (mol%)	69.9	68.9^*b*^	69.0	70.8^*b*^	68.8	70.3

*Strains: *1*, G-1-1-14^T^; *2*, *Azohydromonas ureilytica* UCM-80^*T*^; *3*, *Azohydromonas lata* KACC 15149^*T*^; *4*, *Azohydromonas riparia* UCM-11^*T*^; *5*, *Azohydromonas australica* KACC 15148^*T*^; *6*, “*Azohydromonas aeria*” t3-1-3. All data were obtained from this study, unless otherwise indicated. +, positive; *w*, weak; −, negative; *ND*, data not available. ^*a*^Data from Xue et al. (2020).^*b*^Data from Nguyen and Kim (2017).*

The major cellular fatty acids of strain G-1-1-14^T^ were summed feature 3 (C_16__:__1_*ω*7*c* and/or C_18__:__1_*ω*6*c*), C_16__:__0_, summed feature 8 (C_18__:__1_*ω*7*c* and/or C_18__:__1_*ω*6*c*), and cyclo-C_17__:__0_. Although the major fatty acids of the other type strains of the genus *Azohydromonas* are similar, the presence of minor fatty acids C_14__:__0_ 2-OH, C_15__:__0_ 3-OH, iso-C_15__:__0_ 3-OH, and iso-C_19__:__0_ and the differences of the major and minor fatty acid compositions showed characteristic differences of strain G-1-1-14^T^ compared to other reference cultures (Table 2). The sole respiratory quinone was Q-8, the same as the major respiratory quinone reported for the other members of the genus *Azohydromonas*. The main polar lipids for strain G-1-1-14^T^ were phosphatidylethanolamine (PE), diphosphatidylglycerol (DPG), and phosphatidylglycerol (PG) (Supplementary Figure 8). However, the presence of an unidentified glycolipid (GL), unidentified aminophospholipid (APL), and other minor polar lipids (L1–L4) distinguished strain G-1-1-14^T^ from the other closely related type strains (Supplementary Figure 8; Nguyen and Kim, 2017).

## Conclusion

Based on the above discussed genomic, phylogenetic, phenotypic, and chemotaxonomic characteristic differences, strain G-1-1-14^T^ represents a novel member in the genus *Azohydromonas*, for which the name *Azohydromonas caseinilytica* sp. nov. is proposed.

## Description of *Azohydromonas caseinilytica* sp. nov.

*Azohydromonas caseinilytica* (ca.se.i.ni.ly’ti.ca. N.L. n. *caseinum* casein; Gr. adj. *lytikos* able to dissolve; N.L. fem. adj. *lytica* dissolving; N.L. fem. adj. *caseinilytica* casein-dissolving).

The cells (1.2–1.7 μm wide and 2.6–2.9 μm long) are Gram-stain-negative, non-spore-forming, aerobic, rod-shaped, and motile by means of peritrichous flagella. Colonies on R2A agar are straw white colored, entire, convex, and circular. The colony size of the cells is 1–2 mm on R2A agar for 7 days at 28°C. Cells are positive in the catalase and oxidase tests. The cells grow well on R2A, TSA, and veal infusion agar, while no growth occurs on LBA, BHI, PDA, MA, and marine 2216 agar, but poorly grow on NA. The cells are able to grow autotrophically on hydrogen and fix nitrogen. They grow optimally in the absence of NaCl, but tolerate up to 4.0% (*w*/*v*). The cells grow at 15–45°C (optimum, 25–35°C) and pH 5.5–11.0 (optimum, pH 6.0–8.0) and produce poly 3-hydroxybutyrate. They are able to hydrolyze casein, tyrosine, Tween 60, Tween 40, and aesculin, but not CM-cellulose, starch, chitin, DNA, Tween 80, and urea. Nitrate is reduced, but nitrite is not. Glucose is not fermented. The type strain is positive for acid phosphatase, alkaline phosphatase, cystine arylamidase, esterase lipase (C8), leucine arylamidase, trypsin, valine arylamidase, α-chymotrypsin, α-galactosidase, and β-glucosidase; weakly positive for esterase (C4) and napthol-AS-BI-phosphohydrolase; and is negative for lipase (C14), *N*-acetyl-β-glucosaminidase, α-fucosidase, α-glucosidase, α-mannosidase, β-galactosidase, and β-glucuronidase. The following substrates are assimilated: 3-hydroxybutyric acid, D-glucose, D-mannitol, D-ribose, D-saccharose, lactic acid, L-alanine, L-proline, L-rhamnose, L-serine, *N*-acetyl-glucosamine, potassium gluconate, propionic acid, salicin, and valeric acid. The predominant respiratory quinone is Q-8. The principal cellular fatty acids are summed feature 3 (iso-C_15__:__0_ 2-OH and/or C_16__:__1_*ω*7*c*), C_16__:__0_, summed feature 8 (C_18__:__1_*ω*7*c* and/or C_18__:__1_*ω*6*c*), and cyclo-C_17__:__0_. The major polar lipids are phosphatidylethanolamine, diphosphatidylglycerol, and phosphatidylglycerol. The DNA G + C content of the type strain is 69.9%.

The type strain, G-1-1-14^T^ (= KACC 21615^*T*^ = NBRC 114390^*T*^), was isolated from forest soil, geographically located at Suwon-si, Gyeonggi-do, South Korea (37°18′5″ N and 127°1′56″ E). The GenBank/EMBL/DDBJ accession numbers for the 16S rRNA gene sequence and the whole-genome sequence of strain G-1-1-14^T^ are MN685324 and JABBFW000000000, respectively.

## Data Availability Statement

The datasets presented in this study can be found in online repositories. The names of the repository/repositories and accession number(s) can be found in the article/Supplementary Material.

## Author Contributions

RD and DC conceived, designed, and conducted all the experiments. D-UK interpreted the data. JK coordinated and supervised the study. RD, DC, and D-UK analyzed all the data and prepared the manuscript. All authors read, discussed, edited, and approved the final draft of the manuscript.

## Conflict of Interest

The authors declare that the research was conducted in the absence of any commercial or financial relationships that could be construed as a potential conflict of interest.

## Footnotes

1 https://www.arb-silva.de/aligner/

2 https://crisprcas.i2bc.paris-saclay.fr/

## Supplementary Material

Supplementary Figure 1 Neighbor–joining tree based on 16S rRNA gene sequences showing the phylogenetic position of strain G-1-1-14^T^.

Supplementary Figure 2 Neighbor–joining tree based on 16S rRNA gene sequences showing the phylogenetic position of strain G-1-1-14^T^.

Supplementary Figure 3 Maximum–parsimony tree based on 16S rRNA gene sequences showing the phylogenetic position of strain G-1-1-14^T^.

Supplementary Figure 4 MLSA (Multilocus sequence analyses) tree based on *recA* genes showing the phylogenetic position of strain G-1-1-14^T^ among the closest phylogenetic members.

Supplementary Figure 5 MLSA (Multilocus sequence analyses) tree based on *rpoB* genes showing the phylogenetic position of strain G-1-1-14^T^ among the closest phylogenetic members.

Supplementary Figure 6 Transmission electron microscopic image of strain G-1-1-14^T^ grown on R2A agar at 28°C for 7 days.

Supplementary Figure 7 Subsystem feature counts for strain G-1-1-14^T^ analyzed by RAST (Rapid Annotation using Subsystem technology).

Supplementary Figure 8 Thin-layer chromatograms of the polar lipids from strain G-1-1-14^T^ determined on TLC (thin layer chromatography).

Supplementary Table 1 Average nucleotide identity (ANI) and digital DNA-DNA hybridization (dDDH) of strain G-1-1-14^T^ with reference strains.

Supplementary Table 2 The genes and associated functional roles in strain G-1-1-14^T^ genome revealed by RAST (Rapid Annotation using Subsystem technology) analyses.

Supplementary Table 3 N_2_-fixation regulatory proteins in G-1-1-14^T^ genome.

Supplementary Table 4 The distribution of biosynthetic gene clusters in *Azohydromonas caseinilytica* G-1-1-14^T^.

Supplementary Table 5 Cellular fatty acid profiles (% of totals) of strain G-1-1-14^T^ and other type strains of the genus *Azohydromonas*.
