# Supplementary material for: Azohydromonas caseinilytica sp. nov., a Nitrogen-Fixing Bacterium Isolated From Forest Soil by Using Optimized Culture Method
Source: Front Microbiol. 2021 May 21;12:647132. doi: 10.3389/fmicb.2021.647132 (PMC8175650; doi:10.3389/fmicb.2021.647132)
Supplement: Supplementary Figure 1 — Neighbor–joining tree based on 16S rRNA gene sequences showing the phylogenetic position of strain G-1-1-14T. [file Data_Sheet_1.docx]

Supplementary Material

*Azohydromonas caseinilytica* sp. nov., a nitrogen fixing bacterium isolated from forest soil by using optimized culture method

Ram Hari Dahal^1,2^, Dhiraj Kumar Chaudhary^1,3^, Dong-UK Kim^4^ and Jaisoo Kim^1^*

^1^Department of Life Science, College of Natural Sciences, Kyonggi University, Suwon, Kyonggi-Do 16227, Republic of Korea

^2^Department of Microbiology, School of Medicine, Kyungpook National University, Daegu 41944, Republic of Korea

^3^Department of Environmental Engineering, Korea University Sejong Campus, Sejong City 30019, Republic of Korea

^4^Department of Biological Science, College of Science and Engineering, Sangji University, Wonju, Republic of Korea

*****Correspondence: jkimtamu@kgu.ac.kr; Tel.: +82-31-249-9648


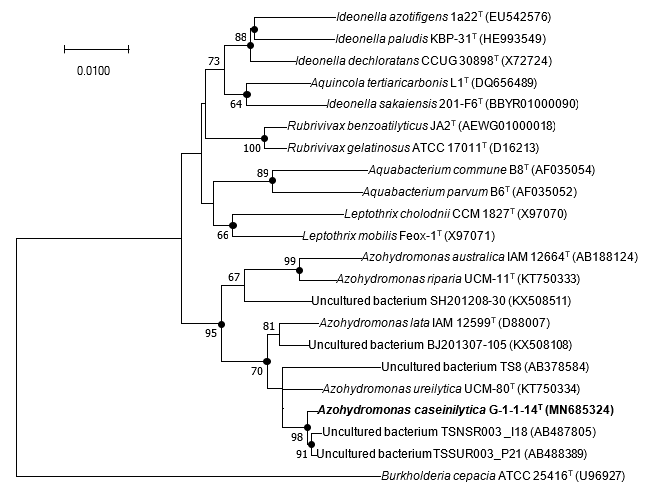


**Supplementary Figure 1.** Neighbour-joining tree based on 16S rRNA gene sequences showing the phylogenetic position of strain G-1-1-14^T^ among closely related members of the order *Burkholderiales* along with previously uncultured bacterial clones. Filled circles indicate nodes recovered by treeing methods (neighbour–joining, maximum–likelihood and maximum–parsimony). The numbers at the nodes indicate the percentage of 1000 bootstrap replicates yielding this topology; only values >50% are shown. *Burkholderia cepacia* ATCC 25416^T^ was used as an out-group. GenBank accession numbers are given in parentheses. Bar, 0.0100 substitutions per nucleotide position.


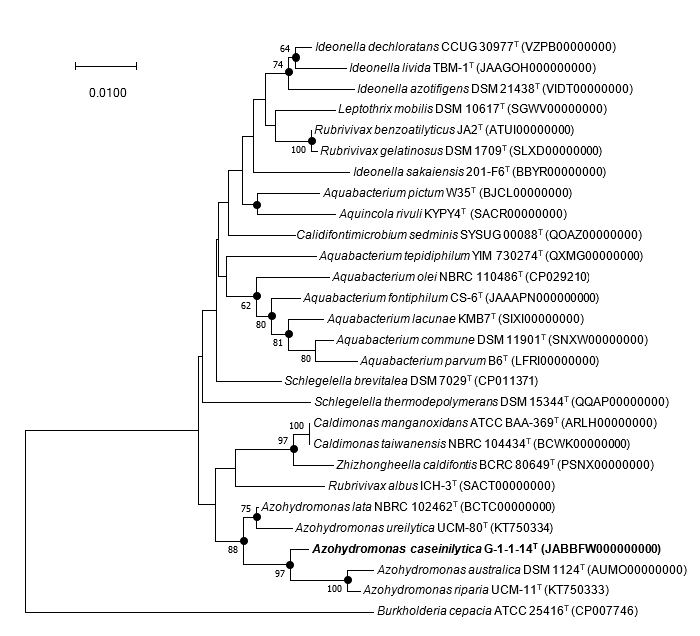


**Supplementary Figure 2.** Neighbour–joining tree based on 16S rRNA gene sequences showing the phylogenetic position of strain G-1-1-14^T^ among closely related members of the order *Burkholderiales*. Filled circles indicate nodes recovered by all three treeing methods (neighbour–joining, maximum–likelihood and maximum–parsimony). The numbers at the nodes indicate the percentage of 1000 bootstrap replicates yielding this topology; only values >50% are shown. *Burkholderia cepacia* ATCC 25416^T^ was used as an out-group. GenBank accession numbers are given in parentheses. Bar, 0.0100 substitutions per nucleotide position.


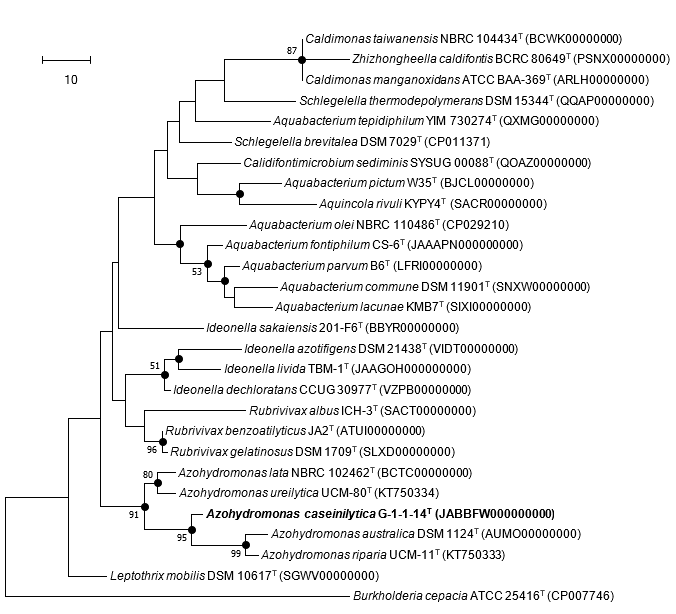


**Supplementary Figure 3.** Maximum–parsimony tree based on 16S rRNA gene sequences showing the phylogenetic position of strain G-1-1-14^T^ among closely related members of the order *Burkholderiales*. Filled circles indicate nodes recovered by all three treeing methods (neighbour–joining, maximum–likelihood and maximum–parsimony). The numbers at the nodes indicate the percentage of 1000 bootstrap replicates yielding this topology; only values >50% are shown. *Burkholderia cepacia* ATCC 25416^T^ was used as an out-group. GenBank accession numbers are given in parentheses. Bar, 10 substitutions per nucleotide position.


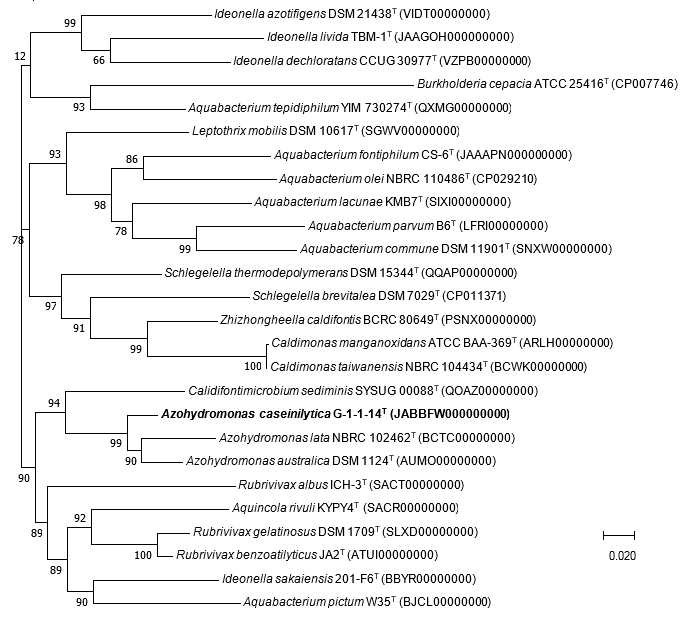


**Supplementary Figure 4.** MLSA (Multilocus sequence analyses) tree based on *recA* genes showing the phylogenetic position of strain G-1-1-14^T^ among the closest phylogenetic members. Bootstrap values are presented at the branch nodes. Bar, 0.020 substitutions per nucleotide position.


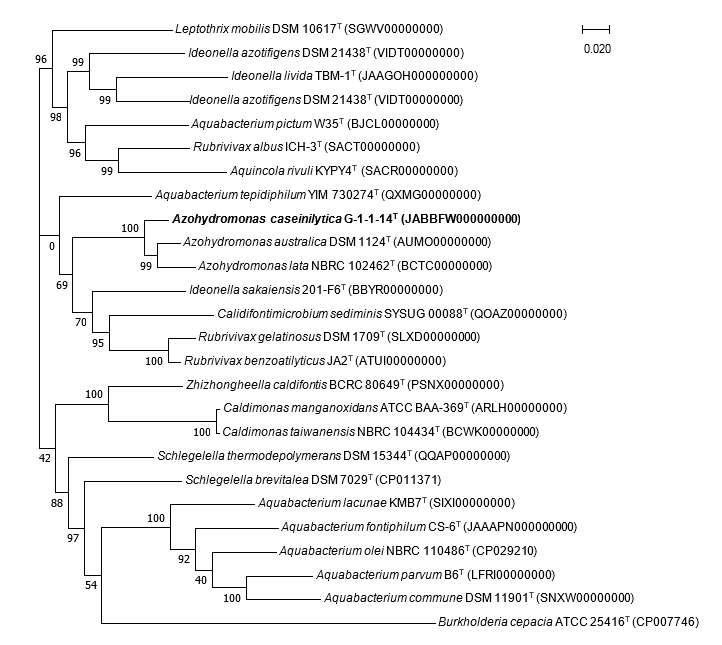


**Supplementary Figure 5.** MLSA (Multilocus sequence analyses) tree based on *rpoB* genes showing the phylogenetic position of strain G-1-1-14^T^ among the closest phylogenetic members. Bootstrap values are presented at the branch nodes. Bar, 0.020 substitutions per nucleotide position.


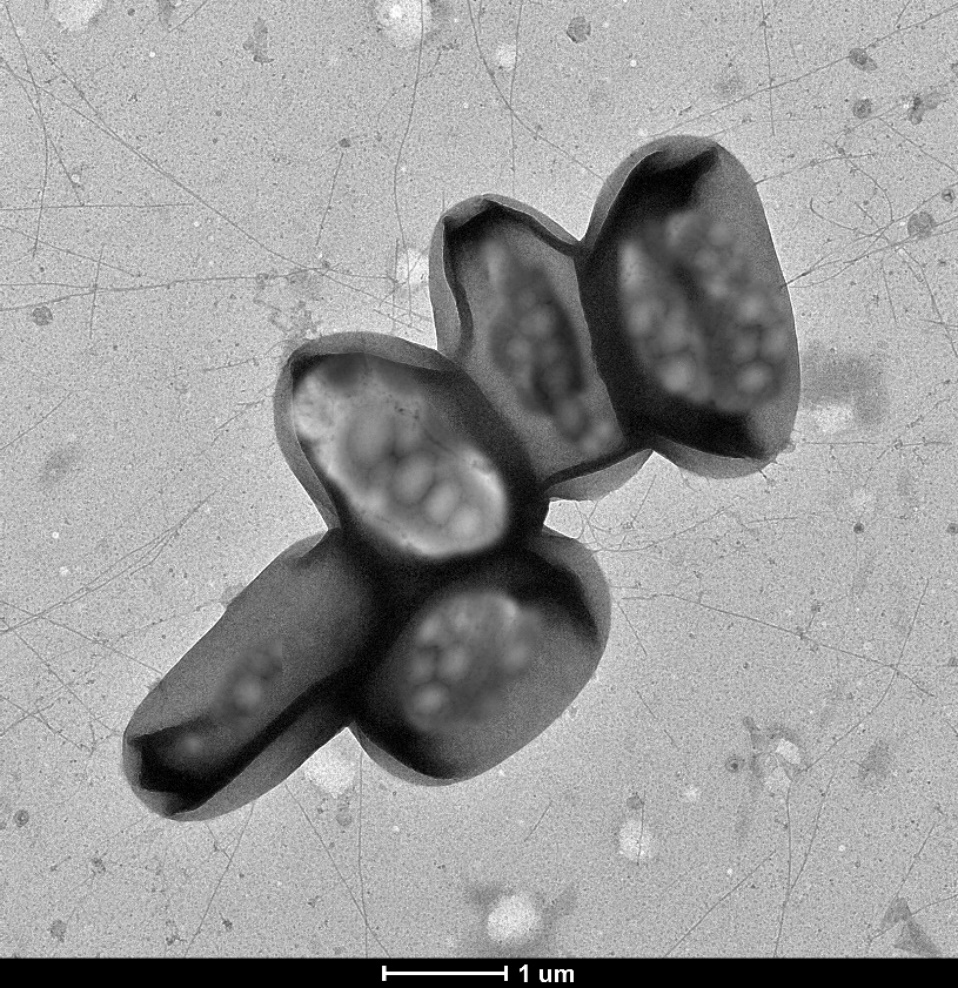


**Supplementary Figure 6.** Transmission electron microscopic image of strain G-1-1-14^T^ grown on R2A agar at 28 °C for 7 days. Bar, 1 µm


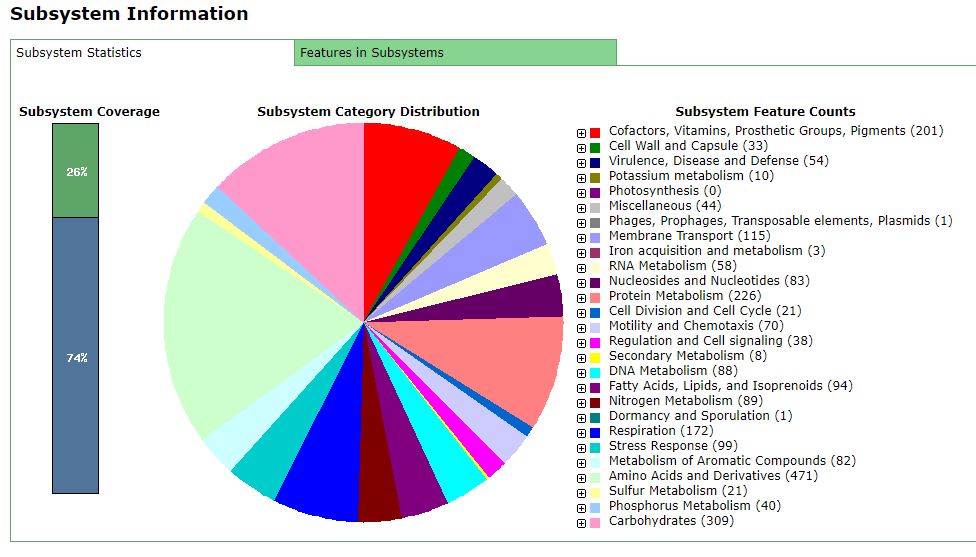


**Supplementary Figure 7.** Subsystem feature counts for strain G-1-1-14^T^ analysed by RAST (Rapid Annotation using Subsystem technology).


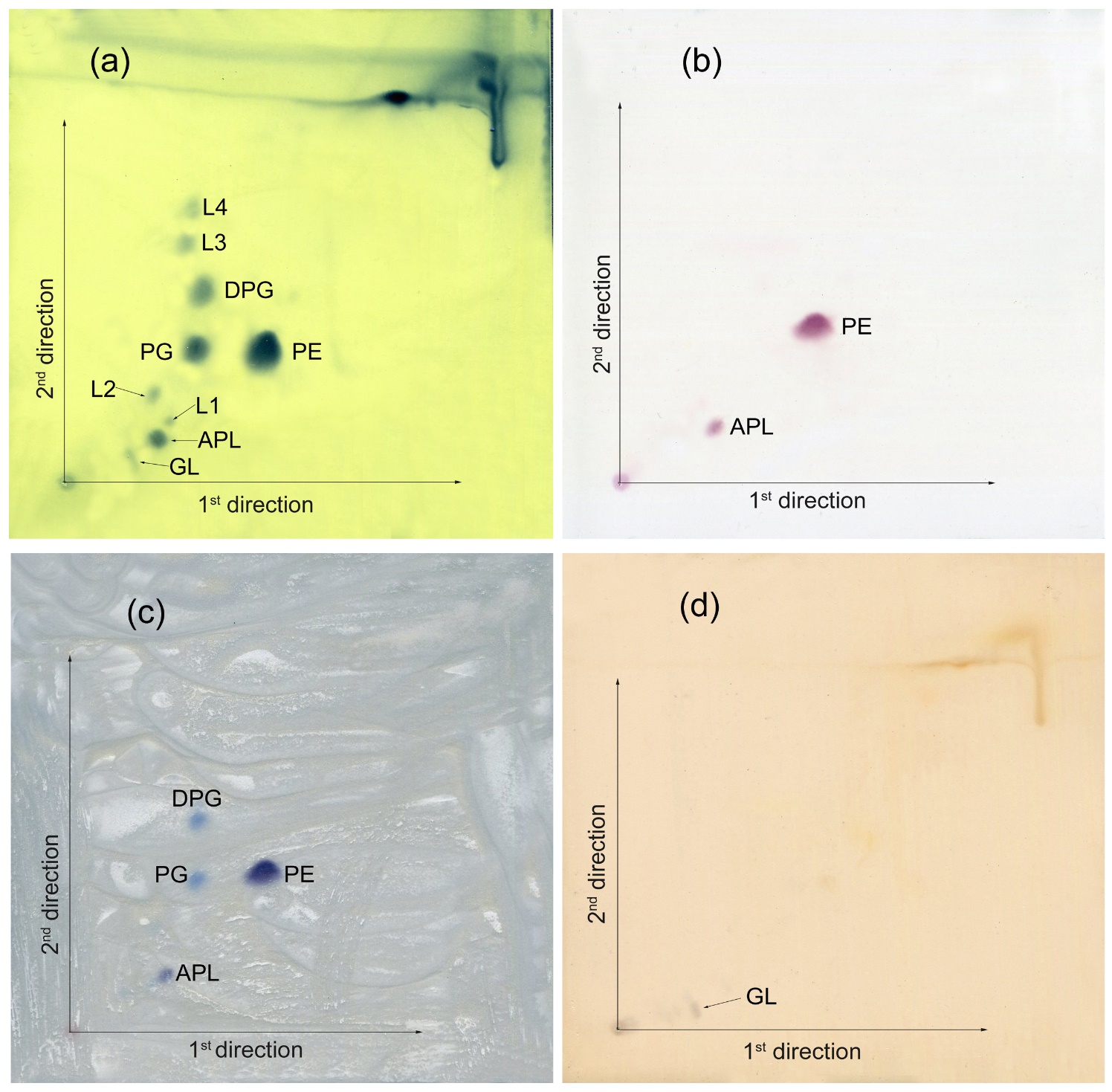


**Supplementary Figure 8.** Thin-layer chromatograms of the polar lipids from strain G-1-1-14^T^ determined on TLC (thin layer chromatography). The components were seen by staining with 5% molybdophosphoric acid in ethanol and heating them to 180 °C for 15 min (a); ninhydrin at 110 °C for 15 min (b); molybdenum at room temperature (c); and *α*-napthol-sulphuric acid at 110 °C for 15 min (d). Abbreviations: PE, phosphatidylethanolamine; DPG, diphosphatidylglycerol; PG, phosphatidylglycerol; GL, unidentified glycolipid; APL, unidentified aminophospholipid; L1–L4, unidentified polar lipids.

**Supplementary Table 1.** Average nucleotide identity (ANI) and digital DNA-DNA hybridization (dDDH) of strain G-1-1-14^T^ with reference strains.

| Strains | G-1-1-14^T^ | | |
| --- | --- | --- | --- |
|  | OrthoANI% |  | dDDH% |
| *Azohydromonas lata* NBRC 102462T | 84.6 |  | 28.6 |
| *Azohydromonas australica* DSM 1124T | 86.5 |  | 31.5 |
| *‘Azohydromonas aeria’* t3-1-3 | 85.3 |  | 29.7 |

**Supplementary Table 2.** The genes and associated functional roles in strain G-1-1-14^T^ genome revealed by RAST (Rapid Annotation using Subsystem technology) analyses.

| Genes | Functional Role |
| --- | --- |
| *nifA* | Nitrogenase (molybdenum-iron)-specific transcriptional regulator NifA |
| *nifB* | Nitrogenase FeMo-cofactor synthesis FeS core scaffold and assembly protein NifB |
| *frdN* | 4Fe-4S ferredoxin, nitrogenase-associated |
| *nifX* | Nitrogenase FeMo-cofactor carrier protein NifX |
| *nifX2* | NifX-associated protein |
| *nifE* | Nitrogenase FeMo-cofactor scaffold and assembly protein NifE |
| *nifN* | Nitrogenase FeMo-cofactor scaffold and assembly protein NifN |
| *nifQ* | Nitrogenase FeMo-cofactor synthesis molybdenum delivery protein NifQ |
| *nifV* | Homocitrate synthase (EC 2.3.3.14) |
| *nifW* | Nitrogenase stabilizing/protective protein NifW |
| *nifH* | Nitrogenase (molybdenum-iron) reductase and maturation protein NifH |
| *nifD* | Nitrogenase (molybdenum-iron) alpha chain (EC 1.18.6.1) |
| *nifK* | Nitrogenase (molybdenum-iron) beta chain (EC 1.18.6.1) |
| *nifZ* | NifZ protein |
| *nifT* | NifT protein |
| *nifO* | Nitrogenase-associated protein NifO |
| Avin2460 | LRV (FeS)4 cluster domain protein clustered with nitrogenase cofactor synthesis |

**Supplementary Table 3.** N_2_-fixation regulatory proteins in G-1-1-14^T^ genome.

| **Accession** | **Contig** | **Locus** | **From** | **To** | **Protein product** | **Length** | **Protein Name** |
| --- | --- | --- | --- | --- | --- | --- | --- |
| [NZ_JABBFW010000002.1](https://www.ncbi.nlm.nih.gov/nuccore/NZ_JABBFW010000002.1) | 2 | *ntrC* | 495336 | 496817 | [WP_169159272.1](https://www.ncbi.nlm.nih.gov/protein/WP_169159272.1) | 493 | Nitrogen regulation protein NR(I) |
| [NZ_JABBFW010000001.1](https://www.ncbi.nlm.nih.gov/nuccore/NZ_JABBFW010000001.1) | 1 | - | 470052 | 470390 | [WP_028997364.1](https://www.ncbi.nlm.nih.gov/protein/WP_028997364.1) | 112 | MULTISPECIES: P-II family nitrogen regulator |
| [NZ_JABBFW010000071.1](https://www.ncbi.nlm.nih.gov/nuccore/NZ_JABBFW010000071.1) | 71 | - | 2456 | 2794 | [WP_169164069.1](https://www.ncbi.nlm.nih.gov/protein/WP_169164069.1) | 112 | P-II family nitrogen regulator |
| [NZ_JABBFW010000008.1](https://www.ncbi.nlm.nih.gov/nuccore/NZ_JABBFW010000008.1) | 8 | - | 236951 | 237358 | [WP_169161004.1](https://www.ncbi.nlm.nih.gov/protein/WP_169161004.1) | 135 | Nitrogen fixation protein NifQ |
| [NZ_JABBFW010000008.1](https://www.ncbi.nlm.nih.gov/nuccore/NZ_JABBFW010000008.1) | 8 | - | 237984 | 238457 | [WP_169161007.1](https://www.ncbi.nlm.nih.gov/protein/WP_169161007.1) | 157 | NifX-associated nitrogen fixation protein |
| [NZ_JABBFW010000008.1](https://www.ncbi.nlm.nih.gov/nuccore/NZ_JABBFW010000008.1) | 8 | *nifX* | 238454 | 238867 | [WP_169161008.1](https://www.ncbi.nlm.nih.gov/protein/WP_169161008.1) | 137 | Nitrogen fixation protein NifX |
| [NZ_JABBFW010000008.1](https://www.ncbi.nlm.nih.gov/nuccore/NZ_JABBFW010000008.1) | 8 | *nifT* | 263908 | 264120 | [WP_169161034.1](https://www.ncbi.nlm.nih.gov/protein/WP_169161034.1) | 70 | Putative nitrogen fixation protein NifT |
| [NZ_JABBFW010000008.1](https://www.ncbi.nlm.nih.gov/nuccore/NZ_JABBFW010000008.1) | 8 | - | 265384 | 265650 | [WP_169161036.1](https://www.ncbi.nlm.nih.gov/protein/WP_169161036.1) | 88 | Nitrogen fixation protein NifZ |
| [NZ_JABBFW010000008.1](https://www.ncbi.nlm.nih.gov/nuccore/NZ_JABBFW010000008.1) | 8 | - | 281902 | 282237 | [WP_169161053.1](https://www.ncbi.nlm.nih.gov/protein/WP_169161053.1) | 111 | Nitrogen fixation protein NifZ |
| [NZ_JABBFW010000009.1](https://www.ncbi.nlm.nih.gov/nuccore/NZ_JABBFW010000009.1) | 9 | - | 54376 | 55188 | [WP_169161139.1](https://www.ncbi.nlm.nih.gov/protein/WP_169161139.1) | 270 | Carbon-nitrogen hydrolase family protein |
| [NZ_JABBFW010000009.1](https://www.ncbi.nlm.nih.gov/nuccore/NZ_JABBFW010000009.1) | 9 | *ptsN* | 145280 | 145750 | [WP_169161211.1](https://www.ncbi.nlm.nih.gov/protein/WP_169161211.1) | 156 | PTS IIA-like nitrogen regulatory protein PtsN |

**Supplementary Table 4.** The distribution of biosynthetic gene clusters in *Azohydromnonas caseinilytica* G-1-1-14^T^ revealed by antiSMASH 5.0. APE Vf, APE Vf biosynthetic gene cluster from *Aliivibrio fischeri* ES114; NRP, non-ribosomal peptide; NRPS, non-ribosomal synthesized peptide; NRPS-like, NRPS -like fragment; NAGGN, *N*-acetylglutaminylglutamine acid; RiPP-like, other unspecified ribosomally synthesised and post-translationally modified peptide product (RIPP) cluster; RRE-containing, RiPP recognition element (RRE) - element containing cluster.

| **Cluster** | **Contig** | **Type** | **From** | **To** | **Most similar known cluster** | **Similarity** | **Core biosynthetic genes** | **Additional biosynthetic genes** |
| --- | --- | --- | --- | --- | --- | --- | --- | --- |
| 1 | 1 | Hserlactone | 398,138 | 418,842 | - | - | 1 | 1 |
| 2 | 2 | Terpene | 298,872 | 320,568 | - | - | 2 | 5 |
| 3 | 6 | RiPP-like | 85,304 | 96,164 | - | - | 1 | 1 |
| 4 | 11 | RRE-containing | 116,078 | 136,347 | Burkholderic acid; NRP + Polyketide:Modular type I | 17% | 1 | 1 |
| 5 | 13 | Terpene | 54,074 | 77,764 | [Carotenoid; terpene](https://mibig.secondarymetabolites.org/go/BGC0000640/1) | 66% | 2 | 2 |
| 6 | 13 | RRE-containing, RiPP-like | 108,265 | 130,418 | - | - | 3 | 1 |
| 7 | 15 | RiPP-like | 74,921 | 85,853 | - | - | 1 | 1 |
| 8 | 25 | NRPS | 38,162 | 82,487 | - | - | 2 | 8 |
| 9 | 28 | NRPS-like | 21,719 | 64,559 | Burkholderic acid; NRP + Polyketide:Modular type I | 6% | 1 | 9 |
| 10 | 32 | Redox-cofactor | 37,760 | 59,950 | - | - | 3 | 3 |
| 11 | 34 | Arylpolyene | 1 | 36,282 | [APE Vf; Other](https://mibig.secondarymetabolites.org/go/BGC0000837/1) | 10% | 1 | 11 |
| 12 | 38 | Lanthipeptide | 24,646 | 42,830 | [Pyoverdin; NRP](https://mibig.secondarymetabolites.org/go/BGC0000413/1) | 1% | 1 | 3 |
| 13 | 41 | NAGGN | 10,213 | 25,010 | - | - | 3 | 3 |

**Supplementary Table 5.** Cellular fatty acid profiles (% of totals) of strain G-1-1-14^T^ and other type strains of the genus *Azohydromonas*.

Strains: 1, G-1-1-14^T^; 2, *Azohydromonas ureilytica* UCM-80^T^; 3, *Azohydromonas lata* KACC 15149^T^; 4, *Azohydromonas riparia* UCM-11^T^; 5, *Azohydromonas australica* KACC 15148^T^; 6, ‘*Azohydromonas areia*’ t3-1-3. All data were obtained from this study unless otherwise indicated. –, not detected.

| **Fatty acid** | **1** | **2** | **3** | **4** | **5** | **6^†^** |
| --- | --- | --- | --- | --- | --- | --- |
| Saturated |  |  |  |  |  |  |
| C_10:0_ | 0.3 | 0.3 | 0.2 | – | 0.2 | – |
| C_12:0_ | 1.9 | 1.1 | 1.6 | 0.7 | 1.9 | 4.6 |
| C_13:0_ | – | – | – | – | 0.2 | – |
| C_14:0_ | 4.1 | 4.4 | 3.9 | 4.0 | 4.2 | 3.2 |
| C_16:0_ | 26.1 | 28.1 | 26.2 | 23.5 | 20.2 | 17.3 |
| C_17:0_ | 0.5 | 0.6 | 1.3 | – | 0.7 | – |
| C_18:0_ | 0.7 | – | 0.7 | 1.1 | 0.6 | 1.1 |
| Unsaturated |  |  |  |  |  |  |
| C_14:1_ *ω*5c | 0.1 | 0.1 | 0.2 | – | – | – |
| C_15:1_ *ω*6c | 0.2 | 0.3 | 0.6 | – | 0.4 | – |
| C_16:1_ *ω*5c | – | 0.3 | – | – | – | – |
| C_17:1_ *ω*6c | – | – | 1.1 | – | – | – |
| C_18:3_ *ω*6c | – | – | – | – | – | 0.8 |
| Hydroxy |  |  |  |  |  |  |
| C_12:0_ 2-OH | 0.9 | 1.5 | 1.1 | 1.9 | 2.0 | – |
| C_14:0_ 2-OH | 0.3 | – | – | 0.6 | – | – |
| C_8:0_ 3-OH | 0.1 | – | – | – | – | – |
| C_10:0_ 3-OH | 4.6 | 2.9 | 2.3 | 3.2 | 2.5 | 4.1 |
| C_12:0_ 3-OH | 0.4 | – | – | – | 2.1 | – |
| C_15:0_ 3-OH | 0.6 | – | – | – | – | – |
| C_16:0_ 3-OH | 0.2 | 0.2 | – | – | – | – |
| iso-C_15:0_ 3-OH | 0.2 | – | – | – | – | – |
| Branched saturated |  |  |  |  |  |  |
| iso-C_15:0_ | – | 0.3 | – | – | – | – |
| iso-C_16:0_ | 0.7 | 0.7 | 0.3 | 0.9 | 0.5 | – |
| iso-C_19:0_ | 0.3 | – | – | – | – | – |
| cyclo-C_17:0_ | 12.7 | 7.3 | 1.1 | 5.9 | 3.7 | 0.9 |
| anteiso-C_15:0_ | – | – | – | 1.6 | – | – |
| Summed features* |  |  |  |  |  |  |
| 3 | 30.6 | 37.3 | 44.5 | 38.3 | 40.0 | 36.8 |
| 8 | 14.5 | 14.6 | 14.9 | 18.3 | 20.8 | 29.7 |

*Summed features represent groups of two or three fatty acids that are treated together for the purpose of evaluation in the MIDI system and include both peaks with discrete ECLs as well as those where the ECLs are not reported separately. Summed feature 3 listed as iso-C_15:0_ 2-OH and/or C_16:1_*ω*7*c* and summed feature 8 listed as C_18:1_*ω*7*c* and/or C_18:1_*ω*6*c*.

^†^Data from Xue *et al.*(Xue et al. 2020).

**References**

Xue H, Piao C gen, Bian D ran, et al (2020) Azohydromonas aeria sp. nov., isolated from air. J Microbiol 58:543–549
